# Exploring Ligand Binding to Calcitonin Gene-Related Peptide Receptors

**DOI:** 10.3389/fmolb.2021.720561

**Published:** 2021-08-26

**Authors:** Giuseppe Deganutti, Silvia Atanasio, Roxana-Maria Rujan, Patrick M. Sexton, Denise Wootten, Christopher A. Reynolds

**Affiliations:** ^1^ Centre for Sport, Exercise and Life Sciences, Coventry University, Coventry, United Kingdom; ^2^ School of Life Sciences, University of Essex, Colchester, United Kingdom; ^3^ Drug Discovery Biology Theme, Monash Institute of Pharmaceutical Sciences, Monash University, Parkville, VIC, Australia; ^4^ ARC Centre for Cryo-Electron Microscopy of Membrane Proteins, Monash Institute of Pharmaceutical Sciences, Monash University, Parkville, VIC, Australia

**Keywords:** GPCR, CGRPR, CGRP, telcagepant, binding, molecular dynamics, supervised molecular dynamics

## Abstract

Class B1 G protein-coupled receptors (GPCRs) are important targets for many diseases, including cancer, diabetes, and heart disease. All the approved drugs for this receptor family are peptides that mimic the endogenous activating hormones. An understanding of how agonists bind and activate class B1 GPCRs is fundamental for the development of therapeutic small molecules. We combined supervised molecular dynamics (SuMD) and classic molecular dynamics (cMD) simulations to study the binding of the calcitonin gene-related peptide (CGRP) to the CGRP receptor (CGRPR). We also evaluated the association and dissociation of the antagonist telcagepant from the extracellular domain (ECD) of CGRPR and the water network perturbation upon binding. This study, which represents the first example of dynamic docking of a class B1 GPCR peptide, delivers insights on several aspects of ligand binding to CGRPR, expanding understanding of the role of the ECD and the receptor-activity modifying protein 1 (RAMP1) on agonist selectivity.

## Introduction

G protein-coupled receptors, the largest family of cell surface receptors (Rosenbaum et al., 2009), are targeted by approximatively 34% of all approved drugs (Hauser et al., 2017). Among the four major classes of vertebrate GPCRs (A, B, C, and F), class B1 are endogenously activated by peptide hormones involved in homeostatic control, e.g., of bone and energy metabolism, and cardiovascular and immune responses (Hollenstein et al., 2014). Drugs acting on class B1 GPCRs could be tremendously useful for the treatment of a range of disorders such as cancer, diabetes, heart disease, hypercalcemia, obesity, and osteoporosis (Hollenstein et al., 2014).

**FIGURE 1:**
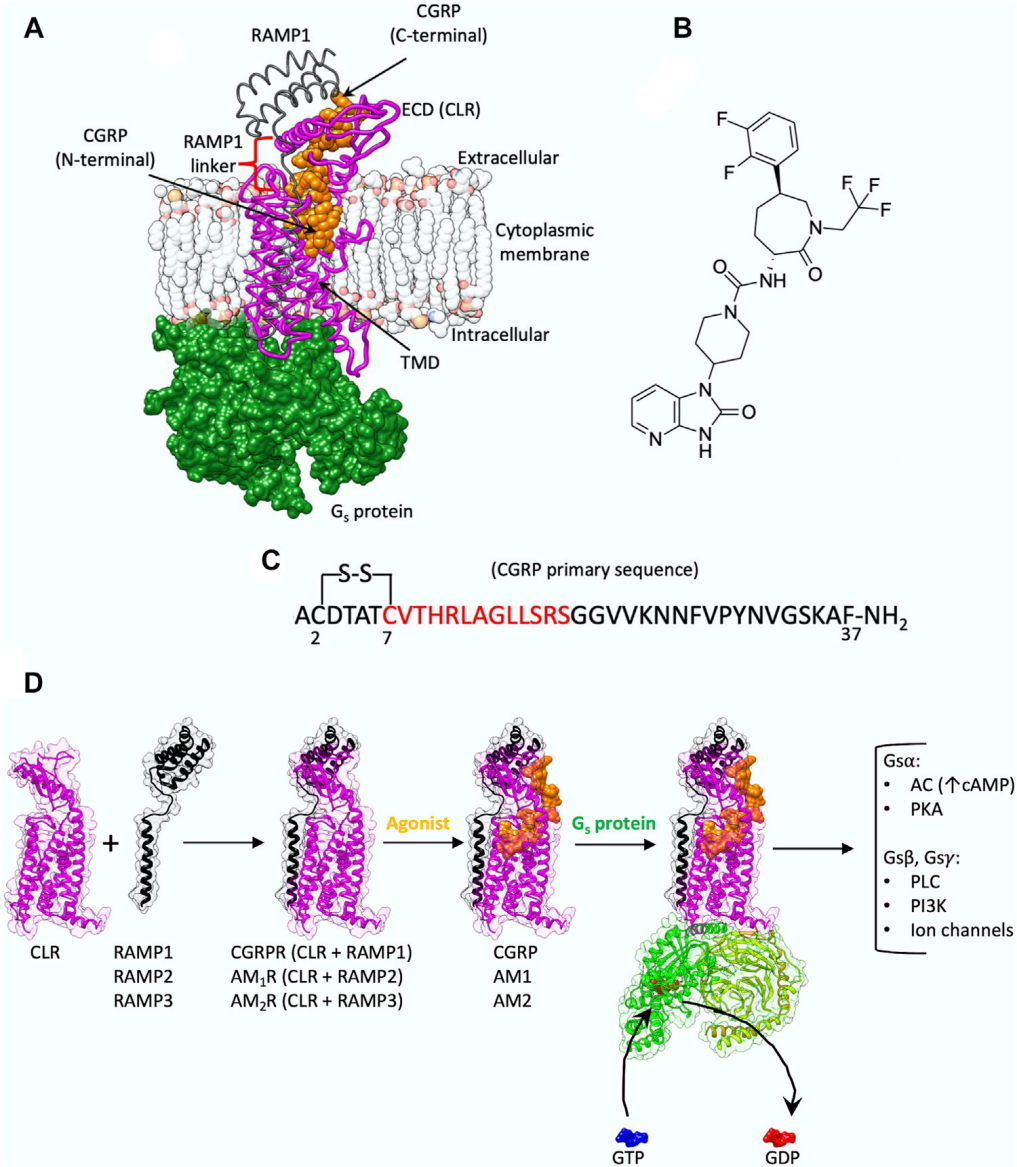
**(A)** CGRPR is a heterodimer membrane protein composed of CLR (purple ribbon) and RAMP1 (gray ribbon); CGRP (orange van der Waals spheres) binds to the TMD and extends towards the ECD. Agonist-bound CGRPR couples to G_s_ protein (green surface). **(B)** Chemical structure of the CGRPR antagonist telcagepant. **(C)** CGRP primary sequence; the residues in the N-terminal helix are indicated in red. **(D)** Schematic representation of the events from the heterodimerization between CLR and RAMPs to G protein signaling. The formation of complexes between CLR and different RAMPs generate receptors with selectivity towards CGRP, AM1 or AM2; upon agonist binding, the trimeric G_s_ protein is recruited and activated, favoring the exchange between GDP and GTP at the level of the G_s_α subunit. After G_s_ subunits dissociation multiple intracellular signaling pathways are triggered (AC, adenylyl cyclase; PKA, protein kinase A; PLC, phospholipase C; PI3K, phosphoinositide 3-kinases).

Since the first active structure, published in 2017 (Liang et al., 2017), the structures of most class B1 GPCRs in the active, G protein complexed, state have been determined through cryo-electron microscopy (cryo-EM), advancing the knowledge of their activation mechanism (García-Nafría and Tate, 2019; Liang et al., 2020b; Ma et al., 2020; Qiao et al., 2020; Liang et al., 2020a; Dong et al., 2020; Duan et al., 2020; Zhou et al., 2020). GPCRs are characterized by a 7-transmembrane helix domain (TMD) and, in class B1, by a N-terminal extracellular domain that contributes to agonist binding, receptor activation, and signaling (de Graaf et al., 2017) (Figure 1A). Heterodimerization with accessory receptor activity-modifying proteins (RAMPs, Figure 1A) can modulate hormone binding and signaling (Hay and Pioszak, 2016) through an allosteric mechanism (Liang et al., 2020a; Pham et al., 2019). The transmembrane spanning RAMPs interact with the calcitonin receptor (CTR) and CTR-like (CLR) receptors at the transmembrane level, making contacts with TM3, TM4 and TM5, and at the extracellular level, where they interface with the ECD (Figures 1A,D). The heterodimer of CLR and RAMP1 displays selectivity for calcitonin gene-related peptide and therefore constitutes the prototypical CGRP receptor (CGRPR, Figures 1A,D) (Liang et al., 2018a). The metastable binding of the neuropeptide CGRP to CGRPR facilitates the intracellular G protein engagement, which in turn allows the peptide to reach the final bound state (Josephs et al., 2021). In this complex, CGRP inserts into the receptor TMD core via a disulfide-bridged N-terminal loop and short amphipathic α-helix, with an unstructured C-terminal tail extended towards the ECD, where it forms an additional anchoring point (Figures 1A,D). The aforementioned stable binding mode can be achieved only after the engagement of the G protein (De Lean et al., 1980; Chung et al., 2011; DeVree et al., 2016), which allosterically facilitates an outward movement of ECL3 and an upward shift of ECL2 (Josephs et al., 2021). The CGRP-bound receptor, prior to G protein coupling, more closely resembles the apo structure with stable binding of the peptide C-terminus to the ECD, and where the peptide N-terminus only transiently and dynamically engaged with the receptor core (Josephs et al., 2021).

CGRP, which is involved in nociceptive transmission and modulation of vasodilatation, is widely expressed in both peripheral and central regions of the nervous systems, including the trigeminal vascular system (Rujan and Reynolds, 2019). Multiple studies have shown that CGRP is associated with migraine pathophysiology (Durham, 2006), making CGRP and its receptor an important target for the treatment of migraine (Rujan and Reynolds, 2019). Despite its involvement in the underlying mechanisms associated with migraine, CGRP also plays a protective role in the cardiovascular system (Kee et al., 2018). Indeed, a novel peptide analogue of CGRP produces a decrease in angiotensin II-induced hypertension as well as a protective effect against heart failure (Aubdool et al., 2017). The heterodimers of CLR and RAMP2 or RAMP3, instead, are more selective for adrenomedullin (AM) and adrenomedullin 2 (AM_2_) peptides and constitute the AM_1_ and AM_2_ receptors, respectively (AM_1_R and AM_2_R, Figure 1D) (Hay and Pioszak, 2016; Hay et al., 2018; Liang et al., 2020a).

The role played by CGRP in migraine has stimulated the pursuit of agents able to block CGRPR activation (Rujan and Reynolds, 2019). Clinical approval of agents targeting this system was first achieved for monoclonal antibodies targeting CGRP or CGRPR. To date, there are three approved monoclonal antibodies against CGRP (galcanezumab, fremanezumab and eptinezumab), and one against CGRPR [erenumab, the first FDA-approved monoclonal antibody targeting a GPCR (Garces et al., 2020)]. Erenumab binds to the ECD of CGRPR, interacting with both CLR and RAMP1 (Garces et al., 2020). The pursuit of orally bioavailable CGRPR antagonists led to the discovery of phenylcaprolactam derivatives. Ubrogepant became the first-in-class oral CGRP antagonist approved for the acute treatment of migraine (December 2019) (Scott, 2020b). This was followed by the approval of rimegepant in February 2020 making it the second approved acute anti-migraine small-molecule drug and the first fast-acting orally disintegrating tablet (Scott, 2020a).

Telcagepant (Figure 1B), a phenylcaprolactam prototype, binds to the ECD of CGRPR with a *K*_*i*_ of 0.8 nM, inhibiting the binding of the CGRP C-terminal domain (Paone et al., 2007; Salvatore et al., 2008; Aksoydan and Durdagi, 2021). CGRP displays biphasic competition with telcagepant (Moore et al., 2009), consistent with the two-step binding mechanism proposed for class B1 GPCR peptides (Pal et al., 2012). According to this model, preliminary interactions between the receptor ECD and the ligand C-terminal domain facilitates the binding of the N-terminal domain within the receptor transmembrane domain (TMD) (Castro et al., 2005; Pal et al., 2012). This increases the local concentration of the N-terminal activation domain of the peptide in the proximity of the target, productively orienting the peptide and favoring the transition of the TMD core from an inactive to an active conformation. The distinct timescales characterizing the two different steps (microsecond and several millisecond, respectively (Castro et al., 2005) suggest that the productive engagement of the peptide N-terminal domain with the receptor TMD core is the rate-limiting step for agonist binding and receptor activation. However, at high concentrations of CGRP, in the presence of telcagepant, monophasic saturation curves are observed (Moore et al., 2009). This is putatively due to an ability of the CGRP N-terminal domain to bind to the TMD of the receptor without the prior formation of the metastable complex between the ECD and the peptide C-terminal domain.

GPCR activation mechanisms also involve water molecules within the TMD (Venkatakrishnan et al., 2019), including the orthosteric (endogenous) ligand binding site (Higgs et al., 2010), and the G protein binding site. From a drug design perspective, discerning whether a region of the binding site is solvated by stable or unstable water molecules can drive the development of tighter or more selective binders. Chemical modifications can be designed to displace unstable water molecules or further stabilize stable ones (Ladbury, 1996; Bortolato et al., 2013).

In the present study, we employed supervised molecular dynamics (Sabbadin and Moro, 2014; Cuzzolin et al., 2016; Salmaso et al., 2017; Deganutti et al., 2020) and classic molecular dynamics simulations to sample the binding of CGRP (Figures 1A,C) and the inhibitor telcagepant (Figure 1B) to CGRPR. The dynamic docking of CGRP to the TMD highlighted residues located within loop 4 of the ECD as a possible key player in the association mechanism. Simulated telcagepant binding and unbinding paths identified residues involved in metastable binding states as well as interactions that could constitute putative kinetic bottlenecks in formation of the high-affinity binding pose. We also simulated the active state CGRPR after removal of any ligand to compare the hydrated regions of the receptor before and after CGRP or telcagepant binding to assess potential changes to CGRPR hydration that occur upon ligand binding.

## Methods

### Telcagepant Force Field Parameters

All the systems (Table 1) were prepared for MD using the CHARMM36 (Huang and MacKerell, 2013; Huang et al., 2017)/CGenFF 3.0.1 (Vanommeslaeghe et al., 2012; Vanommeslaeghe and MacKerell, 2012; Yu et al., 2012) force field combination. The initial telcagepant force field, topology and parameter files were obtained from the ParamChem webserver (Vanommeslaeghe and MacKerell, 2012).

**TABLE 1:** Summary of the MD simulations performed and analyzed.

Ligand	Receptor structure	Simulations performed	# Replicas	Simulation time analyzed (µs)
Telcagepant	CGRPR(CLR:RAMP1) ECD	SuMD binding	4	0.7
Telcagepant	CGRPR(CLR:RAMP1) ECD	SuMD unbinding	5	1.18
		SuMD path sampling		
CGRP	CGRPR(CLR:RAMP1)	SuMD binding	1	1.65
		cMD		
		SuMD path sampling		

### Protein Preparation

In order to speed up simulations involving CGRP, only the G_s_ C-terminal helix (residues N371-L394) of the G protein from the PDB entry 6E3Y was retained, after modelling of the missing segments in the ICL3 (residues 324–328), ECL3 (residues 356–363), ECD (residues 55–63), and CGRP (residues 24–26) as reported in our previous work (Liang et al., 2018a). The resulting active CGRP:CLR:RAMP1 complex was then prepared as follows. Hydrogen atoms were added by means of the pdb2pqr (Dolinsky et al., 2004) and propka (Olsson et al., 2011) software (considering a simulated pH of 7.0); the protonation of titratable side chains was checked by visual inspection. The resulting receptor was inserted in a square 100 Å × 100 Å 1-palmitoyl-2-oleyl-sn-glycerol-3-phosphocholine (POPC) bilayer (previously built by using the VMD Membrane Builder plugin 1.1, Membrane Plugin, Version 1.1. at http://www.ks.uiuc.edu/Research/vmd/plugins/membrane/), through an insertion method (Sommer, 2013). The receptor orientation was obtained by superposing the coordinates on the corresponding structure retrieved from the OPM database (Lomize et al., 2006). Lipids overlapping the receptor transmembrane helical bundle were removed and TIP3P water molecules (Jorgensen et al., 1983) were added to the simulation box by means of the VMD Solvate plugin 1.5 (Solvate Plugin, Version 1.5. at http://www.ks.uiuc.edu/Research/vmd/plugins/solvate/). Finally, overall charge neutrality was reached by adding Na^+^/Cl^−^ counter ions up to the final concentration of 0.150 M), using the VMD Autoionize plugin 1.3 (Autoionize Plugin, Version 1.3. at http://www.ks.uiuc.edu/Research/vmd/plugins/autoionize/).

Simulations involving telcagepant were carried out considering only the CGRPR extracellular domain (CLR residues Q33^ECD^-T131^1.29^, RAMP1 residues E29-P114). Telcagepant was placed about 30 Å away from the ECD and the system was solvated and neutralized as described above.

### System Equilibration and General MD Settings

The MD engine ACEMD (Harvey et al., 2009) was employed for both the equilibration and productive simulations. The equilibration of the CGRP:CLR:RAMP1 complex was achieved in isothermal-isobaric conditions (NPT) using the Berendsen barostat (Berendsen et al., 1984) (target pressure 1 atm) and the Langevin thermostat (Loncharich et al., 1992) (target temperature 300 K) with low damping of 1 ps^−1^. A four-stage procedure was performed (integration time step of 2 fs): first, clashes between protein and lipid atoms were reduced through 2000 conjugate-gradient minimization steps, then a 2 ns long MD simulation was run with a positional constraint of 1 kcal mol^−1^ Å^−2^ on protein and lipid phosphorus atoms. During the second stage, 20 ns of MD simulation was performed constraining only the protein atoms, while in the last equilibration stage, positional constraints were applied only to the protein backbone alpha carbons, for a further 20 ns.

ECD equilibration was achieved in two steps: after 500 cycles of conjugate-gradient minimization, the system was simulated for 5 ns, employing an integration time step of 2 fs, in the isothermal-isobaric conditions (NPT). The system comprising telcagepant and ECD was equilibrated in the NPT ensemble for 2 ns restraining the protein backbone alpha carbons.

Productive trajectories (Table 1) were computed with an integration time step of 4 fs, using the hydrogen mass repartition (Hopkins et al., 2015), in the canonical ensemble (NVT). The target temperature was set at 300 K, using a thermostat damping of 0.1 ps^−1^; the M-SHAKE algorithm (Forester and Smith, 1998; Kräutler et al., 2001) was employed to constrain the bond lengths involving hydrogen atoms. The cut-off distance for electrostatic interactions was set at 9 Å, with a switching function applied beyond 7.5 Å. Long range Coulomb interactions were handled using the particle mesh Ewald summation method (PME) (Essmann et al., 1995) by setting the mesh spacing to 1.0 Å.

### Metadynamics Simulation of CGRP:CLR:RAMP1 Complex

The CGRP:CLR:RAMP1 complex was subjected to a metadynamics simulation to release the agonist from the bound state and relax the ECD in the absence of bound ligand. The well-tempered version of metadynamics (Barducci et al., 2008) was performed employing PLUMED 2.3 (Tribello et al., 2014), biasing the distance between the residues C7-L16 (CGRP) and K134^1.32^-V391^7.64^ (CLR) centroids. Gaussian energy functions were seeded every 1 ps (height = 0.1 kcal/mol, width = 0.1 Å, with a bias factor = 20), at a simulated temperature of 300 K, until the biased distance reached 50 Å. This final frame was used as the starting point for SuMD binding simulations of CGRP.

### The Supervised MD Protocol

The supervised molecular dynamics (SuMD) is an adaptive sampling method (Deganutti and Moro, 2017a) for speeding up simulation of the binding (Cuzzolin et al., 2016; Deganutti et al., 2015; Deganutti and Moro, 2017b; Salmaso et al., 2017; Sabbadin and Moro, 2014; Bower et al., 2018; Bissaro et al., 2019; Bissaro et al., 2020) and unbinding processes (Deganutti et al., 2020). In the first SuMD implementation (Sabbadin and Moro, 2014; Cuzzolin et al., 2016), sampling is gained without the introduction of any energetic bias, by applying a tabu–like algorithm to monitor the distance between the centers of mass (or the geometrical centers) of the ligand and the predicted binding site or the receptor. However, the supervision of a second metric of the system can be considered (Atanasio et al., 2020). A series of short unbiased MD simulations are performed, and, after each simulation, the distances (collected at regular time intervals) are fitted to a linear function. If the resulting slope is negative (for binding), the next simulation step starts from the last set of coordinates and velocities produced, otherwise if the slope is positive, the simulation is restarted by randomly assigning the atomic velocities.

#### Settings for SuMD Binding

The binding of telcagepant to the CGRPR ECD was obtained by supervising the distance between the ligand and CGRPR residue W72^ECD^ (which is roughly in the center of the telcagepant binding site). A series of 500 ps-long time windows were simulated until the distance reached a value of less than 4 Å. Frames were saved every 50 ps and used to interpolate the linear function of the distance during the simulated 500 ps.

To simulate the CGRP binding to CGRPR, the distance between the centroids of residues C7-L16 (CGRP) and K134^1.32^-V391^7.64^ (corresponding to the CLR TMD) was supervised. Four replicas were started from the last frame extracted from the metadynamics, by moving CGRP further away from the receptor and randomly reorienting it. After 250 ns of productive SuMD simulations time, the replica with lower RMSD values to the cryo-EM structure (CGRP residues C7-L16) was used to seed four 1 µs-long classical MD (cMD) simulations. This unsupervised step was performed to facilitate the reorganization of the CGRP:CGRPR metastable states minimizing external influence due to the supervision. The cMD replica characterized by the lowest RMSD value to the cryo-EM structure (CGRP residues C7-L16) was used as a starting point for four additional SuMD binding simulations until visual inspection confirmed appropriate binding of the CGRP N-terminus.

#### Settings for SuMD Unbinding of Telcagepant

The frame with the lowest RMSD value to the X-ray complex 3N7R (ter Haar et al., 2010), representing the ECD in complex with telcagepant, was extracted from SuMD binding trajectories and used as a starting point for CGRPR ECD-telcagepant unbinding simulations (ECD residues Q33^ECD^-T131^1.29^, RAMP1 residues E29-P114). A double supervision was performed: both of the ligand-W72^ECD^ distance and the number of water oxygen atoms within 4 Å of protein atoms and that are hydrogen bonding with telcagepant (if the slope of the linear function plotted on each of the two data series was positive then the time window was productive). The protocol for unbinding differs from the original SuMD binding algorithm, in that the length (Δ*t*) of the short simulations performed increased along the unbinding pathway, according to the formula:

$$\Delta t=\Delta t_{0}Nt_{i}$$ (1)

Δ*t*_0_ is the duration of the very first MD time window and *Nt*_*i*_ represents a factor that is picked from three user-defined values (*Nt*_1_, *Nt*_2_, and *Nt*_3_), according to the last ligand-protein distance detected (Deganutti et al., 2020). Three distance threshold values (*D*_1_, *D*_2_, and *D*_3_) were set and the ligand-protein distance (*r*_*L*_) at the end of each MD run was compared to these threshold values, allowing a decision on the value of the *Nt*_i_ factor according to the following conditions:

$$r_{L}\;\leq \;D_{1}\;\to \;Nt_{i}=\;1$$ (2)

$$D_{1}\;<\;r_{L}\;\leq \;D_{2}\to \;Nt_{i}=\;Nt_{1}$$ (3)

$$D_{2}\;<\;r_{L}\;\leq \;D_{3}\to \;Nt_{i}=\;Nt_{2}$$ (4)

$$D_{3}\;<\;r_{L}\to \;Nt_{i}=\;Nt_{3}$$ (5)

The goal of increasing the simulation time window (Δ*t* in Eq. 1) along the unbinding pathway is to facilitate the sampling of metastable states, which could otherwise be poorly visited. Frames were saved every 50 ps. The initial time window length was 300 ps, with *Nt*_1_, *Nt*_2_, and *Nt*_3_ set to 3, 6, and 10. Values of 5, 8, and 10 Å were used as *D*_1_, *D*_2_, and *D*_3_ distances.

The unbinding was iterated until no ligand-protein van der Waals contact was detected by means of the GetContacts scripts tools (https://getcontacts.github.io). The ligand-protein distance and the number of water oxygen atoms within 4 Å of protein donor/acceptor atoms were computed using PLUMED 2.3. After each productive MD time window, GetContacts was employed to detect and update the protein atoms involved in hydrogen bonds with the ligand, considering a distance of 3.5 Å and an angle value of 120° as geometrical cut-offs. Notably, if no hydrogen bond between the ligand and the protein was present at the end of a productive MD time window, then protein atoms involved in water-mediated or van der Waals interactions were considered.

### SuMD Path Sampling Protocol

SuMD path sampling (Deganutti et al., 2021) (Table 1) was performed considering the output from each SuMD replica, for binding of both CGRP and telcagepant and the unbinding of telcagepant. Each trajectory was aligned on the protein alpha carbon atoms and the frames were clustered according to the ligand RMSD to the starting positions (bin of 1 Å). A frame from each group was randomly extracted and used as a starting point for 20 ns (for telcagepant unbinding) or 30 ns (for CGRPR binding) cMD simulations.

### Analysis of the MD Trajectories

Only the MD trajectories from the SuMD path sampling were analyzed in the case of telcagepant unbinding and CGRPR binding. Interatomic contacts and root mean square deviations (RMSD) were computed using VMD (Humphrey et al., 1996). A contact was considered productive if the distance between two atoms was less than 3.5 Å. Ligand-protein hydrogen bonds were detected using the GetContacts scripts tool (https://getcontacts.github.io), setting a hydrogen bond donor-acceptor distance of 3.5 Å and an angle value of 120° as geometrical cut-offs. Contacts and hydrogen bond persistency are quantified as the percentage of frames (over all the frames obtained by merging the different replicas) in which protein residues formed contacts or hydrogen bonds with the ligand.

Distances between atoms were computed using PLUMED 2.3 (Tribello et al., 2014). The molecular mechanics energy combined with the generalized Born surface area (MM/GBSA) was computed with the MMPBSA.py (Miller et al., 2012) script (AmberTools17 suite at http://ambermd.org/) after transforming the CHARMM psf topology files to an Amber prmtop format using ParmEd (documentation at http://parmed.github.io/ParmEd/html/index.html).

Detection of hydrated spots within CGRPR in the peptide-bound active state (PDB: 6E3Y) was performed on 100 ns long cMD simulations by means of AquaMMapS (Cuzzolin et al., 2018), in the holo state or following removal of CGRP from the complex. This allowed taking into account the flexibility of the systems and thermal fluctuation within the orthosteric binding site.

### Numbering System

Throughout the manuscript, the class B1 GPCR Wootten residue numbering system (Wootten et al., 2013) is displayed as superscripts to the CLR residues numbers.

## Results

### Binding Path of CGRP

The current study focuses on the mechanism of binding for the N-terminal domain of CGRP that is critical to activation of CGRPR. Recent work elucidated the structures of apo and peptide-bound CGRPR in the absence of transducer protein (Josephs et al., 2021) and revealed that, as expected, the peptide C-terminus is stably engaged with the ECD of the receptor, albeit that the ECD is highly dynamic. However, the N-terminus of the peptide only transiently engages with the core of the receptor. As such, there is a gap in understanding of how the CGRP N-terminus (that includes the disulfide-bridged N-terminal loop and short α-helix that extends from this loop) engages with CGRPR to reach the fully-active, G protein-coupled complex that has been experimentally characterized by cryo-EM (Liang et al., 2018a).

**FIGURE 2:**
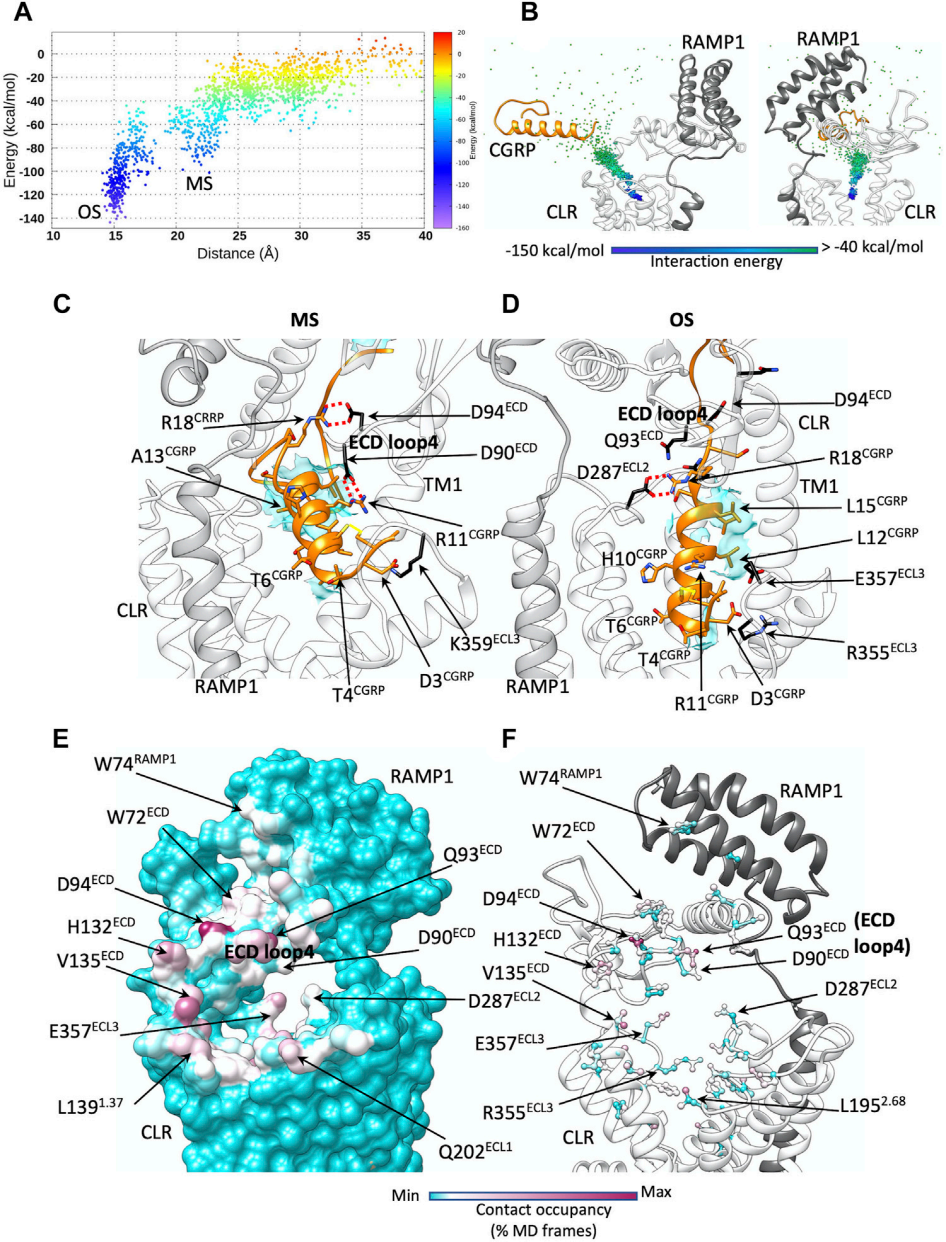
CGRP dynamic docking. **(A)** CGRP binding energy landscape; *x* axes report the distance between the TMD centroid and the CGRP N-terminal helix (the metastable macrostate, MS, is indicated, alongside the deep bound states OS); **(B)** binding path of the centroid of the CGRP N-terminal helix, colored according to the interaction energy with CGRPR; **(C)** representative configuration from the macrostate MS. CGRPR ECD loop 4 forms salt bridges with CGRP R11 and R18; **(D)** representative configuration from the deep bound states OS. Electrostatic interactions are depicted as red dashed lines, while hydrophobic contacts are depicted as cyan transparent surfaces. **(E)** CGRP-CGRPR contacts plotted on CGRPR surface and colored according to the occupancy (% MD frames) of the interactions; **(F)** CGRP-CGRPR contacts plotted on CGRPR atoms and colored according to the occupancy (% MD frames) of the interactions.

During the dynamic docking of the N-terminal helix of CGRP (Supplementary Video S1), the unstructured C-terminal domain spontaneously approached the ECD, forming transitory interactions between F37^CGRP^ and W72^ECD^ or W84^RAMP1^ (Supplementary Table S1). High flexibility of the CGRP C-terminal segment bound to the receptor was also suggested by MD simulations of the active, CGRP:CGRPR:G protein complex (Liang et al., 2018a). These contacts, along with the hydrogen bond between T122^ECD^ backbone and the terminal NH_2_ at F37^CGRP^, are present in the cryo-EM structure and are likely important for the first step of CGRP binding. Very few interactions were formed with RAMP1 (Figures 2C,D; Supplementary Figures S1A,B; Supplementary Tables S1, S2).

CGRP transition from the bulk solvent to the orthosteric site within the TMD was progressively stabilized by interactions of the peptide and receptor (Figures 2A,B). The first contacts between CGRP and CLR were formed at the level of the tunnel entrance shaped by the ECD, TM1, TM2, and ECL1 (Figures 2C–E, Supplementary Figures S1A,B). The peptide disulfide-bridged N-terminal loop (residues 1–7) formed transitory polar interactions with Q33^ECD^, Q93^ECD^, D90^ECD^, N200^ECL1^, N201^ECL1^, and Q202^ECL1^, before V8^CGRP^, L12^CGRP^, and L15^CGRP^ on the hydrophobic side of the amphipathic α-helix of CGRP engaged the side chains of V135^1.33^, L139^1.37^, F142^1.40^, L195^2.68^ in hydrophobic contacts. While the initial polar interactions are not present in the CGRP:CGRPR:G_s_ cryo-EM structure, the latter hydrophobic contacts are reflected in the experimental structure, and throughout the binding simulation acted as a glidant, favoring the peptide insertion into the TMD. While entering the TMD orthosteric site, CGRP formed further interactions with D94^ECD^, F92^ECD^, W354^6.58^, D366^7.39^, and H370^7.43^ (Figures 2C,D; Supplementary Figures S1A,B; Supplementary Tables S1, S2). In the intermediate macrostate, MS (Figure 2A), the agonists sampled several metastable configurations before reaching the bound orthosteric microstate, OS (Figure 2A). This latter state was in remarkable agreement with the experimental coordinates of the fully active complex (Liang et al., 2018a) (Supplementary Video S1). The RMSD of the CGRP Cα carbons to the cryo-EM conformation of CGRP, indeed, reached values lower than 1 Å (3 Å considering the side chain heavy atoms of the peptide helix, Supplementary Figure S2). The agonist residues forming the most frequent interactions during the binding (considering the whole binding trajectory) were D3^CGRP^, T4^CGRP^, T6^CGRP^, T9^CGRP^, H10^CGRP^, R11^CGRP^, L15^CGRP^, R18^CGRP^, and F37^CGRP^ (Supplementary Figures S1C,D; Supplementary Tables S1, S2).

The gradual engagement of the top of TM6, TM7 and ECL3 by the peptide N-terminal domain was accompanied by an inward movement of ECL3 (Supplementary Video S1); ECL3 is important for CGRPR signaling (Barwell et al., 2011) but its dynamics during receptor activation are still unclear. A comparison between the consensus maps of CGRP:CGRPR and CGRP:CGRPR:G_s_ complexes (Josephs et al., 2021) shows that the location of the top of TM6/7/ECL3 in the former structure partially overlaps with the location of the peptide binding pocket in the active CGRPR. Analysis of the conformational dynamics of the CGRP:CGRPR complex revealed that the top of TM6/7/ECL3 dynamically opens and closes in association with transient interaction of the peptide N-terminus and the TMD core (Josephs et al., 2021). This reinforces the concept that the presence of the G protein at the intracellular side of the receptor is needed to allosterically stabilize peptide N-terminal domain binding to the receptor core. Our simulations started from the fully active CGRPR in complex with both CGRP and the Gα helix 5, which could favor the productive engagement of ECL3 with the peptide N-terminus.

The binding energy landscape of CGRP shows low prevalence of states between MS and OS (Figure 2A), indicative of the presence of a transition state. In MS, the positively charged agonist residues R11^CGRP^, R18^CGRP^ formed electrostatic interactions with the side chains of loop 4 residues D90^ECD^ and D94^ECD^ (Figure 2E; Supplementary Figure S1D). In OS, the same CGRP residues interacted with D287^ECL2^, E357^ECL3^ and R355^ECL3^ (Figure 2F; Supplementary Table S2). It follows that during the transition from MS to OS, the ionic network between CGRP and D90^ECD^, D94^ECD^ becomes disrupted to allow new interactions with the TMD.

**FIGURE 3:**
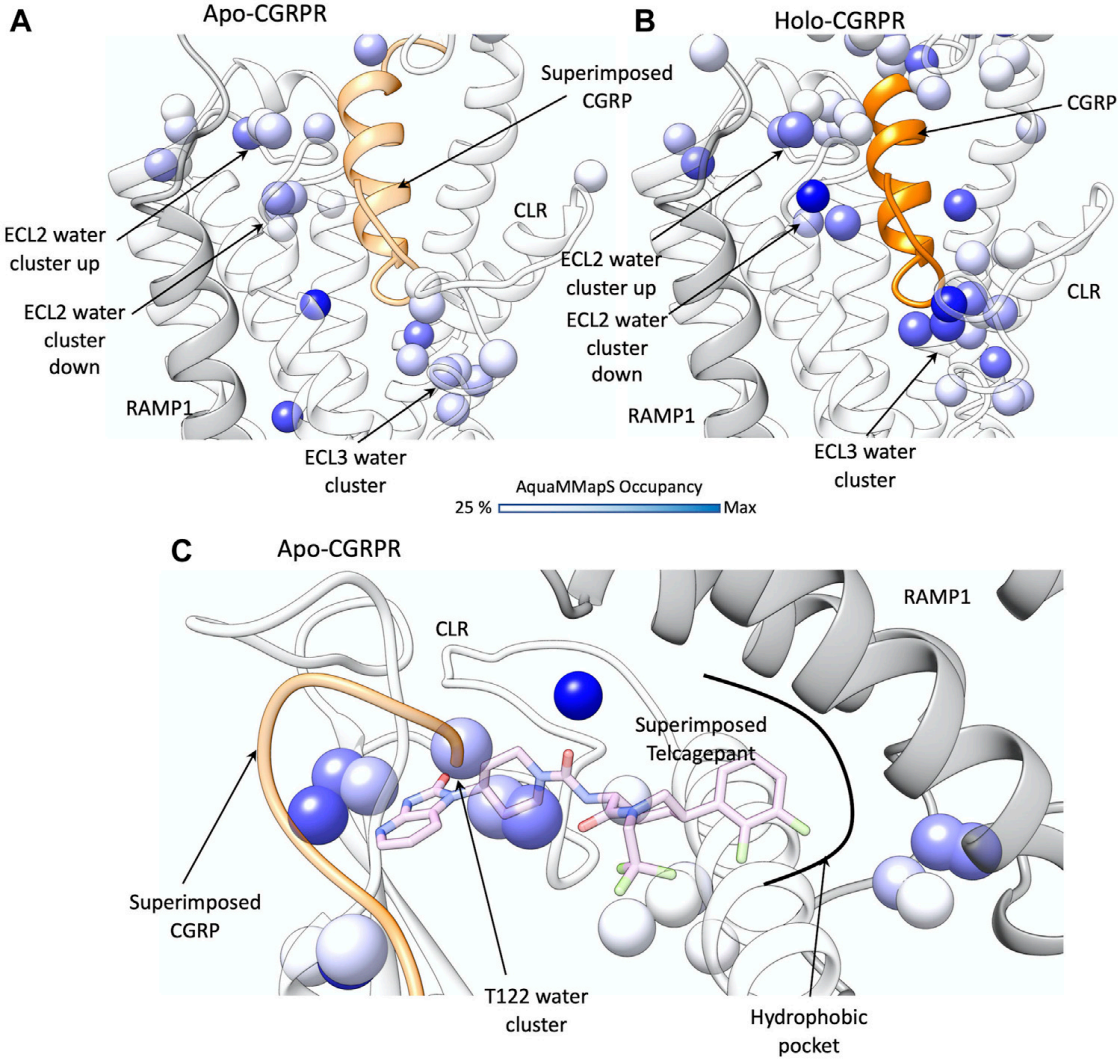
Water network perturbation upon CGRP and telcagepant binding. **(A)** hydration positions in the TMD of the active CGRPR (white ribbon, representative conformation from the first frame of MD simulation), after removal of ligand; CLR and RAMP1 are shown as transparent white and grey ribbons, CGRP from PDB 6E3Y is superimposed (by superimposing TMD scaffolds) and shown as a transparent orange ribbon; **(B)** hydration positions in the holo CGRPR TMD (representative conformation from the first frame of MD simulation). CLR and RAMP1 are shown as transparent white and grey ribbons, CGRP as an orange ribbon; **(C)** hydration positions in the ECD of the active CGRPR, after removal of any ligand; CLR and RAMP1 are shown as transparent white and grey ribbons, CGRP from PDB 6E3Y and telcagepant from PDB 3N7R are superimposed and shown as transparent orange ribbon and pink sticks, respectively. In **(A–C**) the color scale of the hydrated spots corresponds to the occupancy of water molecules during the MD simulation (in dark blue the maximum occupancy).

In the TMD of the active CGRPR, after removal of any ligand (Figure 3A), two hydration clusters were detected in close proximity to ECL2 (namely ECL2 water clusters “up” and “down,” according to the position respective to the backbone of the loop). A further hydrated region was detected near ECL3 (ECL3 water cluster). Two structural water molecules are positioned close to the conserved polar core of the receptor (N187^2.60^ and TM2-TM4 water molecules in Figure 3A); the polar core is important for class B1 receptor activation and biased agonism (Wootten et al., 2013; Wootten et al., 2016; Yin et al., 2017). Upon binding and insertion of the N-terminal helix within the TMD, CGRP stabilizes several water molecules located in proximity to ECL2 and ECL3 (Figure 3B), without overlapping any hydrated region present in the active CGRPR in the absence of ligand. Interactions between CGRPR and the TMD changes the hydration of the TMD by destabilizing the N187^2.60^ and TM2-TM4 water molecules, which are not detected in the peptide-occupied complex (Figure 3B). Overall, these data suggest an efficient solvation profile and formation of a favorable water network during the binding of CGRP.

### Telcagepant Binding and Unbinding Paths

**FIGURE 4:**
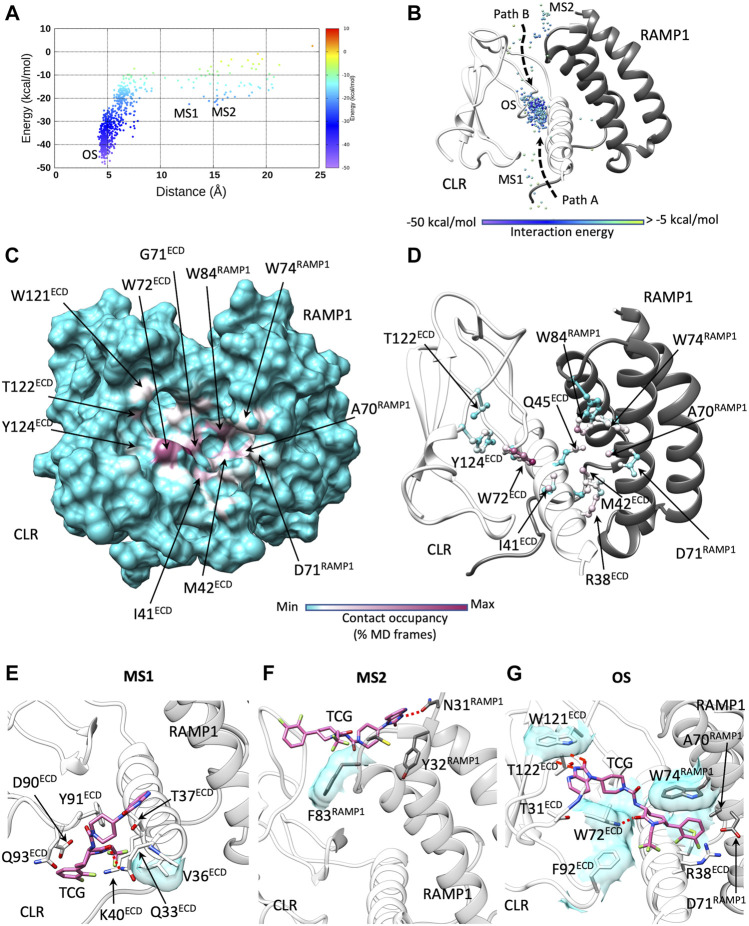
Telcagepant dynamic docking. **(A)** Telcagepant binding energy landscape (the metastable macrostates, MS1 and MS2, are indicated, alongside the bound states, OS, corresponding to the pose in solved structure); **(B)** binding paths of the centroid of telcagepant, colored according to the interaction energy with CGRPR (the metastable macrostates, MS1 and MS2, are indicated, alongside the stable bound states OS); **(C)** Telcagepant-CGRPR contacts plotted on CGRPR surface and colored according to the occupancy (% MD frames) of the interactions; **(D)** Telcagepant-CGRPR contacts plotted on CGRPR atoms and colored according to the occupancy (% MD frames) of the interactions; **(E)** representative configuration from the macrostate, MS1; **(F)** representative configuration from the macrostate, MS2; **(G)** representative configuration from the bound states OS. Electrostatic interactions are depicted as red dashed lines, while hydrophobic contacts are shown as cyan transparent surfaces.

The antagonist telcagepant (Figure 1B) binds into a pocket delimited by the ECD and RAMP1 (ter Haar et al., 2010). During dynamic docking simulations (Figure 4; Supplementary Video S2), the ligand rapidly reached the crystallographic conformation following two possible paths (Paths A and B in Figure 4B) and forming intermediate metastable states in correspondence of macrostates, denoted MS1 and MS2 (Figures 4A,B). The most persistent contacts with the receptor were formed around the binding site (Figures 4C,D; Supplementary Tables S3, S4) and comprised interactions with side chains of W72^ECD^, I41^ECD^, M42^ECD^, R38^ECD^, Q45^ECD^, W74^RAMP1^, and W84^RAMP1^, which are also involved in the final bound state. Besides the hydrogen bonds with W72^ECD^ and the backbone of T122^ECD^, the antagonist formed transitory polar interactions with N128^ECD^, R119^ECD^, and W121^ECD^ (Supplementary Figure S3; Supplementary Table S4). Binding Path A was facilitated by interactions with the ECD helix 1 residues Q33^ECD^, V36^ECD^, T37^ECD^, K40^ECD^ (Figure 4E). Binding Path B involved interactions with the RAMP1 residues N31^RAMP1^ and F83^RAMP1^, located over the binding site (Figure 4F). Consistent with mutagenesis studies (Moore et al., 2010), no significant hydrogen bonds were formed between telcagepant and R67^RAMP1^, D71^RAMP1^, and E78^RAMP1^ (Supplementary Table S4).

**FIGURE 5:**
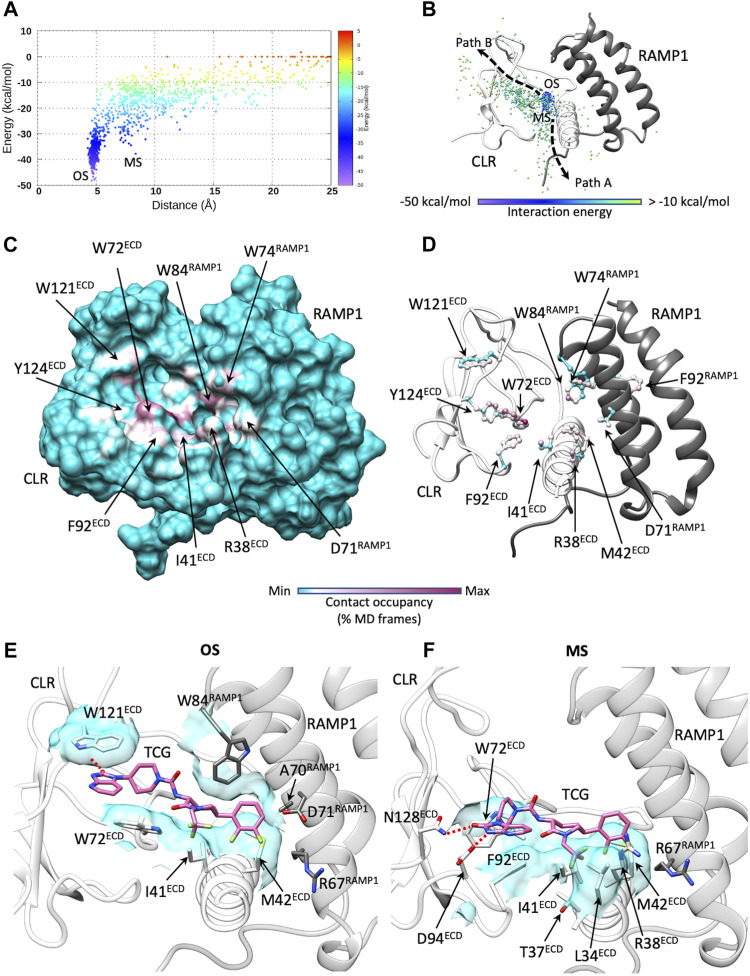
Telcagepant dynamic unbinding. **(A)** Telcagepant unbinding energy landscape (the metastable macrostates, MS, are indicated, alongside the stable bound states OS); **(B)** unbinding paths of the centroid of telcagepant, colored according to the interaction energy with CGRPR (the metastable macrostates, MS, are indicated, alongside the stable bound states, OS); **(C)** Telcagepant-CGRPR contacts plotted on CGRPR surface and colored according to the occupancy (% MD frames) of the interactions; **(D)** Telcagepant-CGRPR contacts plotted on CGRPR atoms and colored according to the occupancy (% MD frames) of the interactions; **(E)** representative configuration from the stable bound state, OS; **(F)** representative configuration from the metastable macrostate, MS. Electrostatic interactions are depicted as red dashed lines, while hydrophobic contacts are shown as cyan transparent surfaces.

Unbinding simulations (Figure 5; Supplementary Figure S4; Supplementary Tables S5, S6) sampled one notable metastable macrostate (MS in Figures 5A,B) close to the bound configuration observed in the static crystallographic structure (ter Haar et al., 2010) (OS in Figures 5A,B). The transition between OS and MS took place in the early stages of ligand dissociation and involved the rupture of the hydrogen bonds with the backbone of T122^ECD^ in favor of new transient interactions with D94^ECD^ and N128^ECD^ (Figures 5E,F). After release from the hydrophobic interactions between the 1,2-dichlorobenzene group and W74^RAMP1^, W84^RAMP1^, and M42^ECD^, telcagepant followed two possible unbinding routes from MS (Figure 5B): unbinding Path A roughly represented the inverse of binding Path A, while unbinding Path B followed an alternative direction, away from RAMP1, which involved interactions with W72^ECD^.

The ECD is the first site of stable contacts for class B1 peptides during the proposed two-stage peptide agonist binding (Hoare, 2005), as well as the target of the antagonist telcagepant (Figure 3C). In the active CGRPR, following removal of the ligand, AquaMMapS analysis highlighted two clusters of stable water molecules in the proximity of T122^ECD^ (Figure 3C), within the binding site of telcagepant and the distal residues of the CGRP C-terminal domain. The backbone of T122^ECD^ forms hydrogen bonds with either the primary amide of the CGRP C-terminal residue F37^CGRP^ or with the phthalimide moiety of telcagepant. It follows that both ligands likely displace stable water molecules to form hydrogen bonds with the T122^ECD^ backbone. Upon binding, telcagepant extends the difluorophenyl moiety into a hydrophobic pocket and displaces unstable water molecules, as suggested by the absence of hydrated spots in this region of ECD (Figure 3C).

## Discussion

Recently resolved class B1 GPCR structures reveal a heterogenous conformational landscape that involves the extracellular elements of the receptor (Liang et al., 2020b; Zhang et al., 2020). For example, different glucagon-like peptide receptor (GLP-1R) agonists imprint divergent orientations of the ECD and alternative conformations of the top of TM1, TM6, TM7, ECL2, and ECL3. Some of these structural features, such as an outward conformation of ECL3, appear linked to biased agonism of different ligands (Liang et al., 2018b; Zhang et al., 2020).

The structural mechanism underlining the agonist two-step binding and class B1 receptor activation is still unclear. The recent structures of CGRPR in apo and CGRP-bound (before G protein coupling) forms have delivered new insights into the transition from the resting to the fully active state of the receptor (Josephs et al., 2021). Interestingly, in the consensus cryo-EM maps only minor conformational changes in the backbone of the receptor were observed before and after the binding of CGRP. Given the intrinsic flexibility that characterizes GPCRs (Latorraca et al., 2017), orthogonal methods such as hydrogen-deuterium exchange mass spectrometry (Yang et al., 2015; Josephs et al., 2021), 3D variance analysis of cryo-EM conformational continuums (Liang et al., 2020a; Dong et al., 2020; Zhang et al., 2020; Josephs et al., 2021) and molecular dynamics simulations (Deganutti et al., 2019) are required to characterize CGRPR molecular motions and advance the understanding of its pharmacology.

The adaptive sampling protocol employed in the current study focused on the peptide N-terminal approach to the CGRPR TMD, and therefore did not interrogate the preliminary interactions between the ECD and the peptide C-terminal domain. The simulation of the complete two-stage binding mechanism requires a more thorough computational sampling and should take into account the secondary structure characterizing the class B1 peptides in solution before any interaction with the ECD occurs. GLP-1, secretin (Sec), glucagon (GCG), pituitary adenylate cyclase activating polypeptide (PACAP), vasoactive intestinal polypeptide (VIP), parathyroid hormone 1 (PTH1), and corticotrophin-releasing factor (CRF) bind with an extended α-helix conformation spanning from the TMD to the ECD binding sites. However, in solution, many of them present a disordered N-terminal segment (Gronenborn et al., 1987; Neidigh et al., 2001). This suggests that the formation of the α-helix is a hallmark of the binding. On the other hand, agonists from the calcitonin sub-family, such as CGRP, in solution show only partial folding of the N-terminal in α-helix motif (Breeze et al., 1991) and engage their target by retaining this structural organization.

This structural conservation between solution and receptor-bound CGRP structures rules out any major conformational changes of the CGRP during the binding, reinforcing our findings. RAMPs allosterically alter the dynamics of the ECD, ECL2 and ECL3 and the signaling profile of CLR, in a peptide-dependent manner (Liang et al., 2020a). However, the structural reasons for the binding selectivity exerted on CLR is still unclear. We speculate that different RAMPs may modulate the affinity of CLR agonists by driving divergent metastable states of ECD loop 4 during binding to the TMD. The membrane-proximal linker region of the RAMP1 (residues 102–118, Figures 1A, 2D,E) is an important contributor to the allosteric modulation of CLR (Liang et al., 2020a). The different rotameric states of D113^RAMP1^ in the apo and CGRP-bound states of CGRPR (Josephs et al., 2021) along with dynamic differences in this region reported for the RAMPs (Supplementary Figure S5) suggests that the flexibility of the linker could play a more complex role in selectivity of agonist binding. The position of ECD loop 4 in the CGRP:CGRPR:G_s_, AM:AM_1_R:G_s_, AM:AM_2_R:G_s_ and AM2:AM_2_R:G_s_ complexes (Liang et al., 2020a) is different, implying a possible correlation with the linker region. The exchange of the RAMP1 linker with the RAMP2 linker produces a loss of CGRP potency ascribable to a correlated dynamic between the ECD and the G protein (Liang et al., 2020a).

Our simulations showed that ECD loop 4 may act as a selectivity filter for the incoming agonist. The shape and dynamics of the gate formed by ECD, TM1, TM2 and ECL1 appear to be linked to ECD mobility and, in turn, to the interactions between the RAMP linker and CLR. We speculate that different intramolecular interactions with CLR differently modulate ECD flexibility. These differences in the dynamics of loop 4 residues D90^ECD^, Q93^ECD^, and D94^ECD^ could contribute to the selectivity displayed by CLR in complex with the RAMPs, thus influencing the overall kinetics of binding.

We cannot rule out an involvement of the extracellular surface of the membrane during the first steps of the association between the N-terminal domain of CGRPR and TMD extracellular vestibule. However, the preliminary binding of the peptide C-terminus to the ECD should restrain the path of the N-terminal domain, reducing the possibility of diffusion from the membrane.

Simulations of telcagepant highlighted key features and a partial overlap of association and dissociation paths from the ECD. The telcagepant binding site is easily accessible from the bulk solvent without hindrances and presents stable water molecules only in the proximity of T122^ECD^, which the phthalimide moiety is likely to occupy without unfavorable energy contributions thanks to the hydrogen bonds formed by the amide group. It follows that desolvation of the pocket should be ruled out as a bottleneck to binding (Sykes et al., 2019), consistent with the “fast-on” nature of telcagepant. Our simulations also suggest that the low-nanomolar/high picomolar affinity of telcagepant for CGRPR is due to the fast association rather than a long residence time, which is reported to be close to 2 min (Moore et al., 2009).

In summary, we propose an updated scenario for the binding to CGRPR by the endogenous agonist and a prototypic antagonist. Exploiting the recent structural information on different states of the CGRPR (Josephs et al., 2021) and GLP-1R in the absence of endogenous agonist bound (Wu et al., 2020), future work will be directed to establishing MD protocols able to reconstruct the different phases of the proposed two-step binding mechanism of all class B peptides, taking into account the conformational transition occurring upon binding.

## Data Availability

SuMD simulations dataset can be download from the Zenodo repository at https://zenodo.org/record/5109537.
