# Supplementary material for: Exploring Ligand Binding to Calcitonin Gene-Related Peptide Receptors
Source: Front Mol Biosci. 2021 Aug 26;8:720561. doi: 10.3389/fmolb.2021.720561 (PMC8427520; doi:10.3389/fmolb.2021.720561)
Supplement: Supplementary file 1 [file DataSheet1.PDF]

Supplementary Material

Video S1. CGRP binding to CGRP. Two side views of the dynamic docking of CGRP (orange) to CGRPR (CLR in white, RAMP1 in grey) from SuMD for N-terminal peptide binding to the CGRPR core. Residues in contact along the trajectory are shown as sticks; hydrogen bonds between side chains are depicted as dashed red lines. The simulation time indicated is cumulative of SuMD and classic MD simulations.

Video S2. Telcagepant binding to CGRP ECD. Telcagepant (magenta stick representation) binds to the CGRPR ECD reaching the experimentally observed conformation (transparent green lines). Residues in contact along the trajectory are shown as sticks; hydrogen bonds between side chains are depicted as dashed red lines. The simulation time indicated is cumulative of five SuMD replicas.

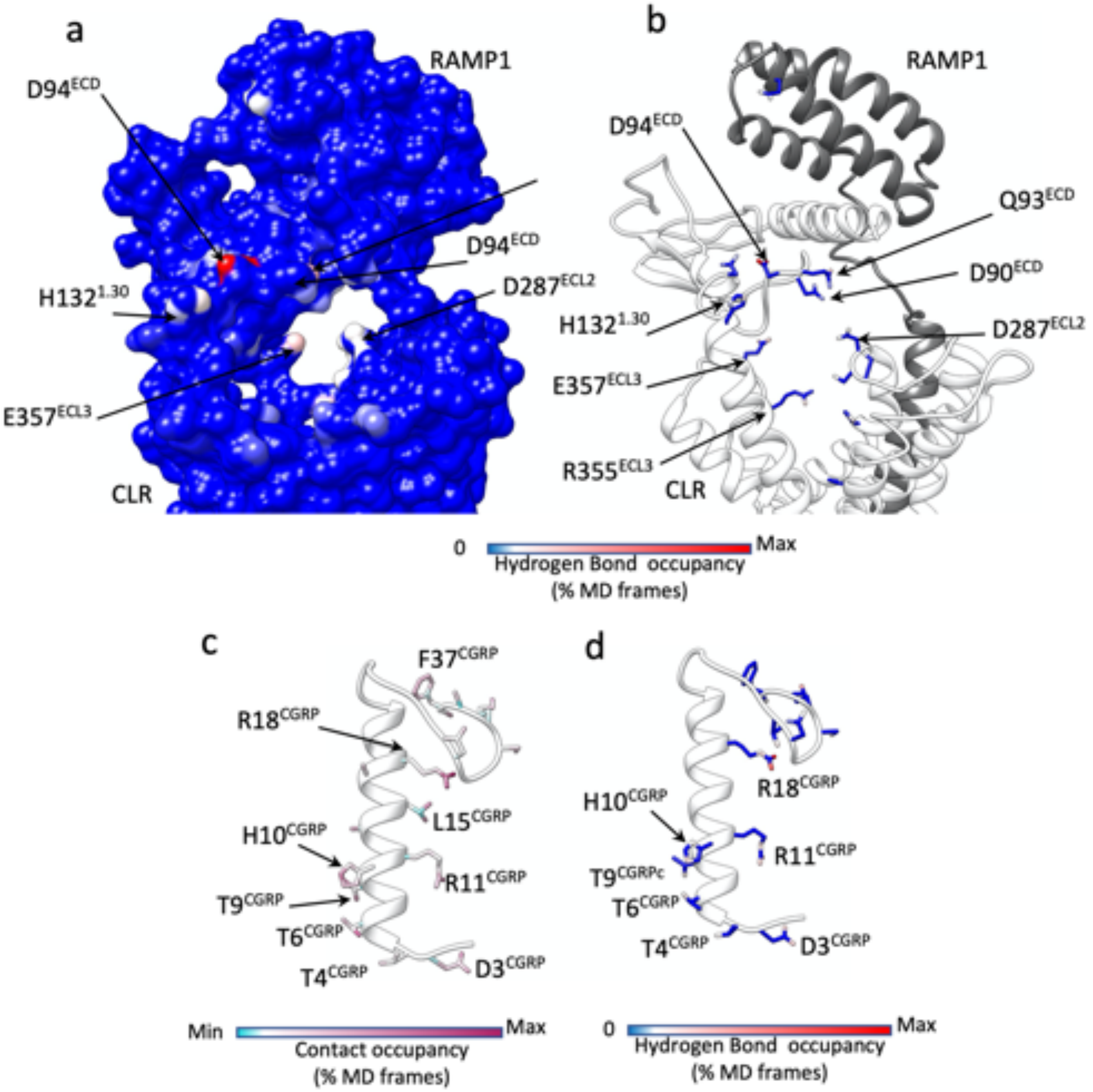

Figure S1. **a)** CGRP-CGRPR hydrogen bonds during SuMD N-terminal peptide binding path sampling, plotted on CGRPR surface and colored according to the occupancy (% MD frames) of the interaction; **b)** CGRP-CGRPR hydrogen bonds during SuMD binding path sampling, plotted on CGRPR atoms and colored according to the occupancy (% MD frames) of the interaction; **c)** CGRP residues most frequently involved in contacts with CGRPR, colored according to the occupancy (% MD frames) of the interaction; **d)** CGRP residues most frequently involved in hydrogen bonds with CGRPR, colored according to the occupancy (% MD frames) of the interaction.

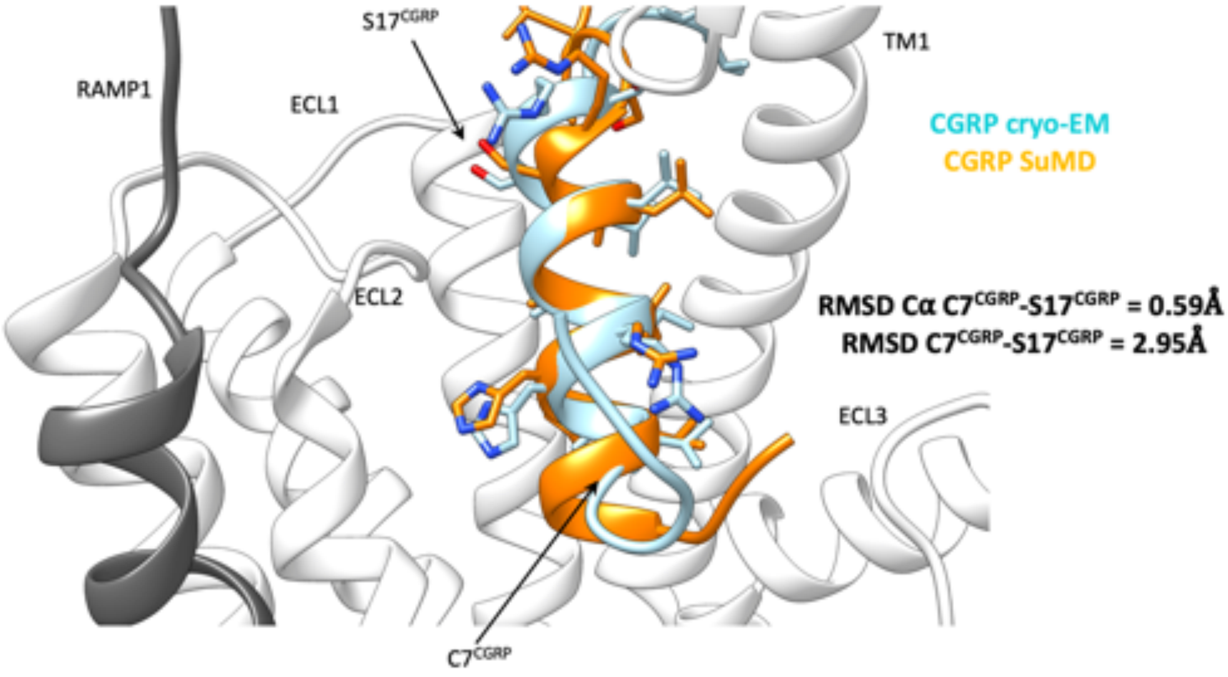

Figure S2. Superposition between CGRP in the cryo-EM structure 6E3Y (cyan) and a representative MD frame from SuMD binding path sampling.

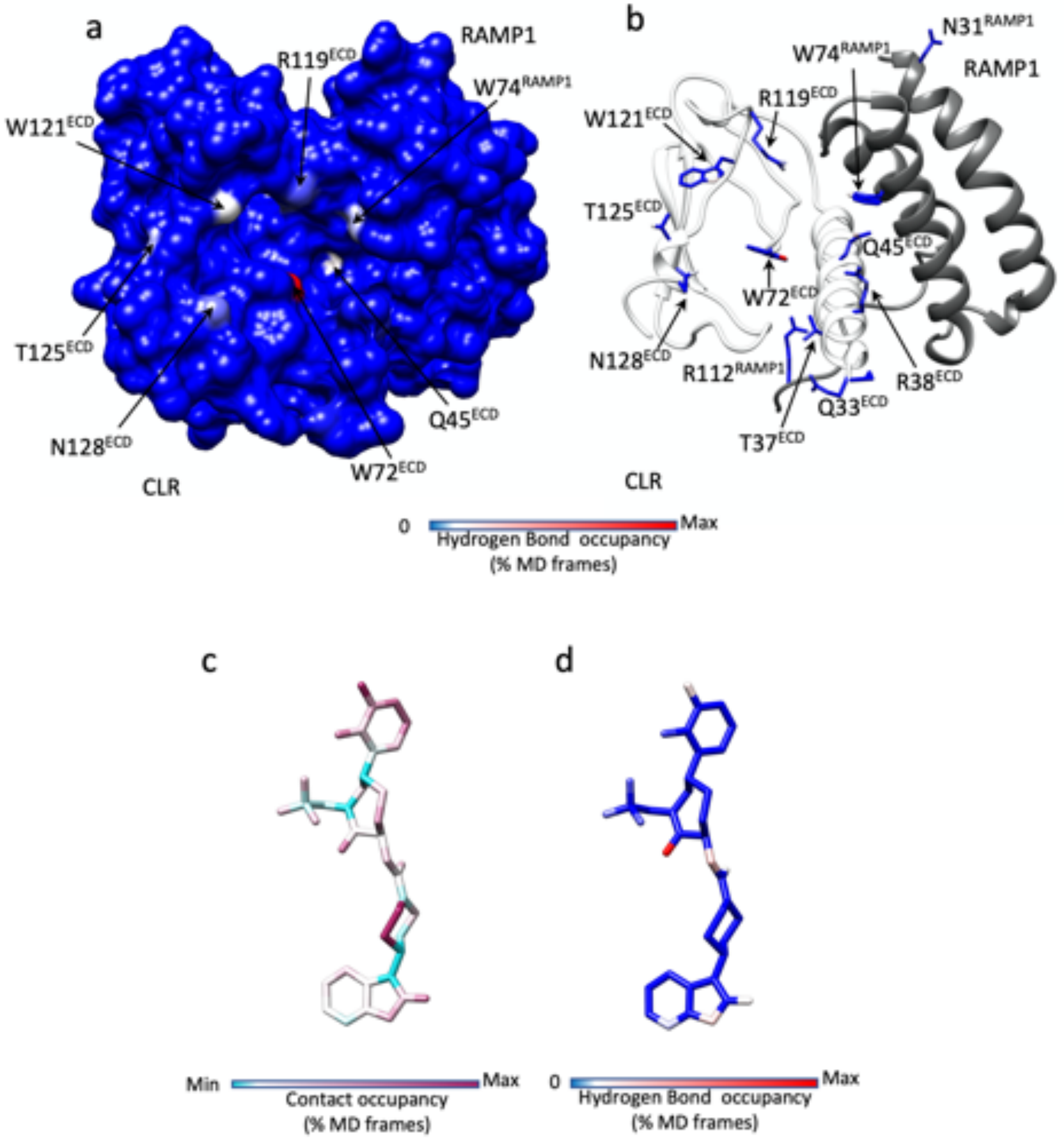

Figure S3. **a)** Telcagepant-CGRPR hydrogen bonds during SuMD binding path sampling, plotted on CGRPR surface and colored according to the occupancy (% MD frames) of the interaction; **b)** telcagepant-CGRPR hydrogen bonds plotted on CGRPR atoms and colored according to the occupancy (% MD frames) of the interaction; **c)** telcagepant atoms most frequently involved in contacts with CGRPR, colored according to the occupancy (% MD frames) of the interaction; **d)** telcagepant atoms most frequently involved in hydrogen bonds with CGRPR, colored according to the occupancy (% MD frames) of the interaction.

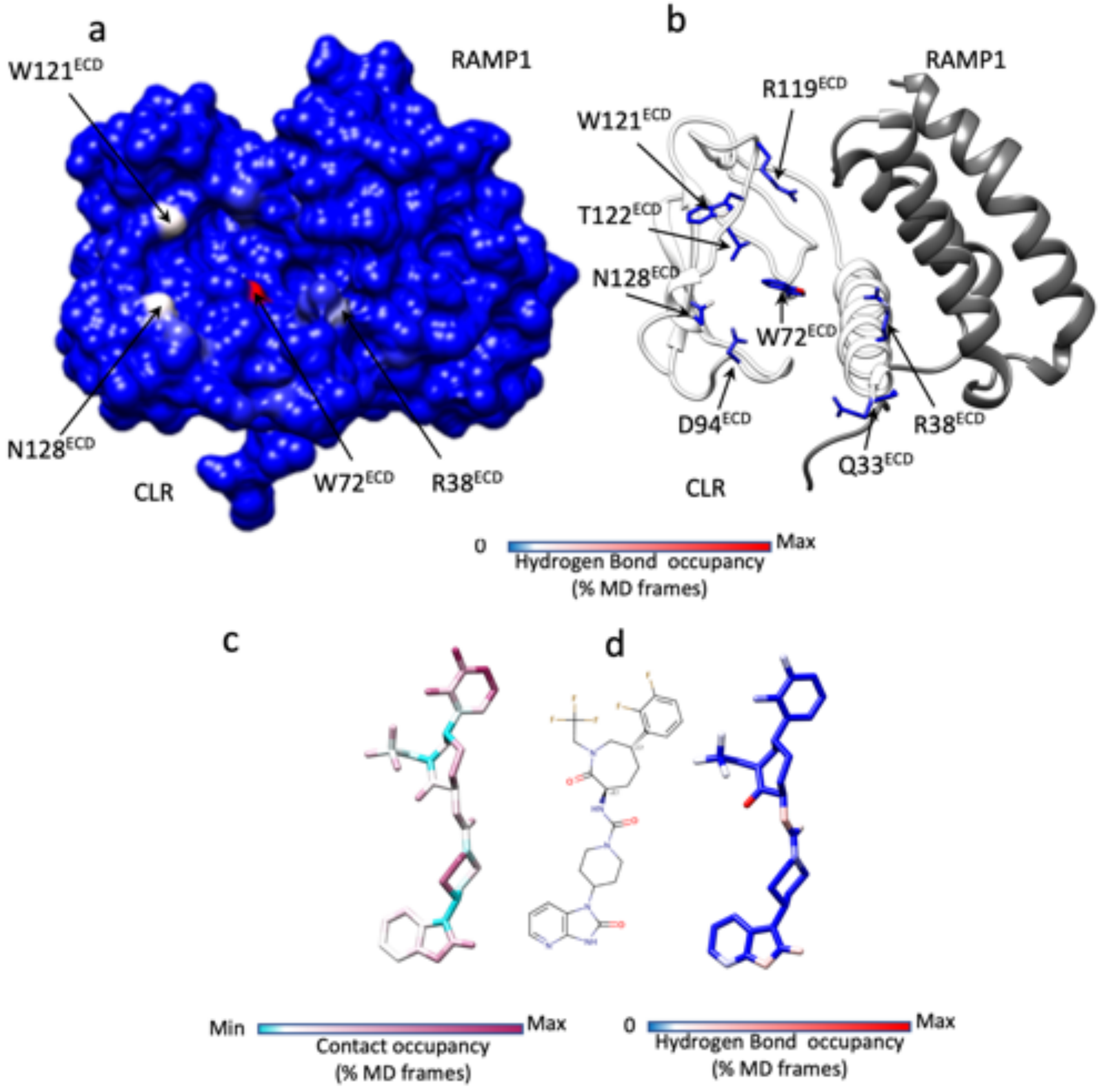

Figure S4. **a)** Telcagepant-CGRPR hydrogen bonds during SuMD unbinding path sampling, plotted on CGRPR surface and colored according to the occupancy (% MD frames) of the interaction; **b)** telcagepant-CGRPR hydrogen bonds during SuMD unbinding path sampling, plotted on CGRPR atoms and colored according to the occupancy (% MD frames) of the interaction; **c)** telcagepant atoms most frequently involved in contacts with CGRPR, colored according to the occupancy (% MD frames) of the interaction; **d)** telcagepant atoms most frequently involved in hydrogen bonds with CGRPR, colored according to the occupancy (% MD frames) of the interaction.

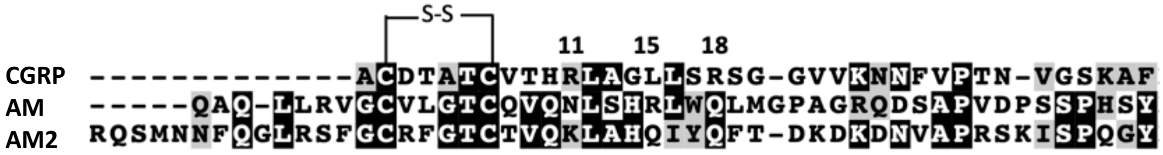

Figure S5. Multiple sequence alignment of CGRP, AM and AM2.

Table S1. Contacts between CGRP and CGRPR during SuMD binding path sampling simulations. Contact persistency is quantified as the percentage of frames (over all the frames obtained by merging the different replicas) in which protein residues were closer than 3.5 Å to CGRP. Interactions present in the final bound state are in bold.

CGRPR Residue	CGRP Residue	Contact occupancy (% MD frames)
Q93^{ECD}	R11	43.3
D94^{ECD}	R18	41.5
V135$^{1.33}$	L15	36.4
**F142$^{.40}$**	**L12**	**32**
E357^{ECL3}	A1	30.6
L139$^{1.37}$	L12	27.4
**L195$^{2.68}$**	**T9**	**26.7**
**Q202^{ECL1}**	**S17**	**26.5**
**Q93^{ECD}**	**R18**	**25.7**
L195$^{2.68}$	A5	25.6
A138$^{1.36}$	V8	22.9
Q33^{ECD}	H10	22.5
M373$^{7.46}$	D3	22.3
**W72^{ECD}**	**F37**	**21.8**
**R355^{ECL3}**	**A1**	**21.4**
**R355^{ECL3}**	**D3**	**21.1**

**V135$^{1.33}$**	**R11**	**21**
Q93^{ECD}	H10	20.7
**S286^{ECL2}**	**H10**	**19.1**
H132$^{1.30}$	L15	18
**M223$^{3.40}$**	**T6**	**17.7**
**A138$^{1.36}$**	**L12**	**17.4**
**Y227$^{3.44}$**	**A5**	**17.2**
A199^{ECL1}	T9	17.1
**Q202^{ECL1}**	**G21**	**17.1**
**A138$^{1.36}$**	**L15**	**16.8**
**H295$^{5.40}$**	**T6**	**15.8**
M373$^{7.46}$	T4	15.4
D366$^{7.39}$	A1	15
E357^{ECL3}	R11	15
H370$^{7.43}$	D3	15
**L195$^{2.68}$**	**L12**	**15**
D94^{ECD}	K35	14.9
**H194$^{2.67}$**	**T9**	**14.9**
**T191$^{2.64}$**	**T9**	**14.7**
**W354$^{6.58}$**	**D3**	**14.3**

**A199^{ECL1}**	**A13**	**14.1**
A199^{ECL1}	A5	14
**V198$^{2.71}$**	**A13**	**14**
F142$^{1.40}$	T9	13.4
A199^{ECL1}	T6	13.2
M369$^{7.42}$	D3	13.2
**M373$^{7.46}$**	**A5**	**13.2**
H132$^{1.30}$	R18	13.1
V135$^{1.33}$	L12	12.9
Q93^{ECD}	L15	12.6
**Y227$^{3.44}$**	**T6**	**12.5**
L139$^{1.37}$	F27	12
**H295$^{5.40}$**	**H10**	**11.9**
H132$^{1.30}$	T30	11.7
L139$^{1.37}$	L15	11.7
**W72^{ECD}**	**A36**	**11.6**
Y227$^{3.44}$	T4	11.5
**L195$^{2.68}$**	**L16**	**11.4**
**V198$^{2.71}$**	**S17**	**11.2**
F349$^{6.53}$	D3	11

A138$^{1.36}$	R11	10.8
R38^{ECD}	F37	10.6
D90^{ECD}	A1	10.6
**Q202^{ECL1}**	**G20**	**10.6**
Q93^{ECD}	G14	10.6
F92^{ECD}	R18	10.5
**V198$^{2.71}$**	**L16**	**10.5**
Q202^{ECL1}	L16	10.4
W84^{RAMP1}	F37	10.3
H132$^{1.30}$	F37	10.2
P97^{ECD}	R11	10.2
**H194$^{2.67}$**	**A13**	**10**

Table S2. Hydrogen bonds between CGRP and CGRPR during SuMD binding path sampling simulations. Hydrogen bond persistency is quantified as the percentage of frames (over all the frames obtained by merging the different replicas) in which protein residues were closer than 3.5 Å to CGRP. Interactions present in the final bound state are in bold.

**CGRPR Residue**	**CGRP Residue**	**Hydrogen Bond occupancy (% MD frames)**
D94^{ECD}	R18	40.3

**R355^{ECL3}**	**D3**	**18.3**
E357^{ECL3}	R11	14.9
Q93^{ECD}	R11	12.6
D94^{ECD}	K35	10.8
E78^{RAMP1}	K35	8.5
S285^{ECL2}	D3	7.1
**H295$^{5.40}$**	**T6**	**6.8**
H132$^{1.30}$	R18	6.6
T191$^{2.64}$	T9	6.3
Y227$^{3.44}$	T4	6.1
H295$^{5.40}$	H10	5.9
**D287^{ECL2}**	**R18**	**5.8**
S286^{ECL2}	H10	5.7
K40^{ECD}	D3	5.6
**D96^{ECD}**	**R11**	**5.2**
Q93^{ECD}	R18	5.1
Q33^{ECD}	H10	5
H132$^{1.30}$	T30	5
**K359$^{7.32}$**	**D3**	**4.4**
D94^{ECD}	S34	4.3

**D94^{ECD}**	**T30**	**4.2**
W354$^{6.58}$	D3	3.6
D90^{ECD}	R11	3.3

Table S3. Contacts between telcagepant and CGRPR during SuMD binding path sampling simulations. Contact persistency is quantified as the percentage of frames (over all the frames obtained by merging the different replicas) in which protein residues were closer than 3.5 Å to telcagepant. Interactions present in the final bound state are in bold.

CGRPR Residue	Contact occupancy (% MD frames)
**W72^{ECD}**	**88.4**
**W84^{RAMP1}**	**81.4**
**G71^{ECD}**	**78.9**
**I41^{ECD}**	**73.3**
**R38^{ECD}**	**72.2**
**M42^{ECD}**	**58**
**W74^{RAMP1}**	**52.3**
**A70^{RAMP1}**	**48.4**
**Y124^{ECD}**	**45.9**
R67^{RAMP1}	39.1
D71^{RAMP1}	37

P85^{RAMP1}	34.1
**T122^{ECD}**	**33.5**
F92^{ECD}	33
**W121^{ECD}**	**31.2**
D70^{ECD}	25.7
L34^{ECD}	25.1
Q45^{ECD}	20.1
R119^{ECD}	18.4
T37^{ECD}	14.6
F93^{RAMP1}	12.9
Q33^{ECD}	12.7

Table S4. Hydrogen bonds between telcagepant and CGRPR during SuMD binding path sampling simulations. Hydrogen bond persistency is quantified as the percentage of frames (over all the frames obtained by merging the different replicas) in which protein residues were closer than 3.5 Å to telcagepant. Interactions present in the final bound state are in bold.

CGRPR Residue	Hydrogen Bond occupancy (% MD frames)
**W72^{ECD}**	**71.9**
Q45^{ECD}	11.8
**W121^{ECD}**	**9.9**

**W74^{RAMP1}**	**7.1**
T125^{ECD}	6.6
R119^{ECD}	4.9
N128^{ECD}	4.3
**R38^{ECD}**	**2.3**
Q33^{ECD}	0.8
N31^{RAMP1}	0.7
R112^{ECD}	0.3
T37^{ECD}	0.3

Table S5. Contacts between telcagepant and CGRPR during SuMD unbinding path sampling simulations. Contact persistency is quantified as the percentage of frames (over all the frames obtained by merging the different replicas) in which protein residues were closer than 3.5 Å to telcagepant. Interactions present in the initial bound state are in bold.

CGRPR Residue	Contact occupancy (% MD frames)
**W72^{ECD}**	**62.6**
**Y124^{ECD}**	**49.5**
**R38^{ECD}**	**47.8**
F92^{ECD}	46.6
**I41^{ECD}**	**43.5**

**W84^{RAMP1}**	**39.6**
**W74^{RAMP1}**	**37.4**
**G71^{ECD}**	**37.3**
**M42^{ECD}**	**31.5**
**W121^{ECD}**	**30.6**
**T122^{ECD}**	**30.5**
D71^{RAMP1}	30.4
**A70^{RAMP1}**	**29.7**
R67^{RAMP1}	26.3
F95^{ECD}	24.4
L34^{ECD}	23.9
T37^{ECD}	16
N128^{ECD}	15.4
P85^{RAMP1}	11.1
D94^{ECD}	10.7

Table S6. Hydrogen bonds between telcagepant and CGRPR during SuMD unbinding path sampling simulations. Hydrogen bond persistency is quantified as the percentage of frames (over all the frames obtained by merging the different replicas) in which the hydrogen bond was present. Interactions present in the initial bound state are in bold.

CGRPR Residue	Hydrogen Bond occupancy

	**(% MD frames)**
**W72^{ECD}**	**34.9**
**W121^{ECD}**	**7.1**
N128^{ECD}	6.7
R38^{ECD}	5.1
D94^{ECD}	3.2
